# Radiological Landmarks for Joint Line Level in Challenging Total Ankle Arthroplasty

**DOI:** 10.3390/jcm13154451

**Published:** 2024-07-29

**Authors:** Simone Ottavio Zielli, Antonio Mazzotti, Elena Artioli, Alberto Arceri, Federico Sgubbi, Laura Langone, Pejman Abdi, Cesare Faldini

**Affiliations:** 1 1st Orthopaedics and Traumatologic Clinic, IRCCS Istituto Ortopedico Rizzoli, 40136 Bologna, Italy; simoneottavio.zielli@ior.it (S.O.Z.); antonio.mazzotti@ior.it (A.M.); alberto.arceri@ior.it (A.A.); pejman.abdi@ior.it (P.A.);; 2 Department of Biomedical and Neuromotor Sciences (DIBINEM), Alma Mater Studiorum University of Bologna, 40123 Bologna, Italy

**Keywords:** total ankle replacement, revision arthroplasty, custom-made, patient specific instrumentation

## Abstract

**Background:** Although Total Ankle Arthroplasty (TAA) is primarily performed for post-traumatic ankle arthritis with joint disruption, anatomical landmarks for Joint Line (JL) level are typically preserved. However, severe Post-Traumatic Bone Loss (PTBL) or TAA revision may render some landmarks unidentifiable, challenging JL restoration. **Methods:** Patients undergoing customized TAA for severe PTBL or revision were enrolled. Custom-made implants, based on 3D CT scans, were designed to address bone defects and provide adequate bone support. Evaluated parameters, measured on bilateral ankle weight-bearing radiographs taken preoperatively and 6–8 months postoperatively, included JL Height Ratio (JLHR) and the distances from JL to the Lateral Malleolus apex (LM-JL), the posterior colliculus of the Medial Malleolus (MM-JL), and the Gissane Calcaneal Sulcus (CS-JL). Reproducibility and variability were assessed, and comparisons were made between radiological parameters measured at TAA and those at the contralateral ankle. **Results:** Thirteen patients were included. Intra- and interobserver reliability demonstrated excellent values. The least variability was observed in the LM-JL distance. Statistically significant correlations were found between CS-JL and MM-JL distances in the operated limb and between the CS-JL of the operated limb and the contralateral ankle. While TAA parameters did not show statistically significant differences compared with the contralateral ankle, a trend toward proximalization of the JL was noted. **Conclusions:** This study demonstrated good reproducibility of the analyzed parameters for evaluating JL in TAA among patients with severe PTBL or undergoing revision surgery. However, these parameters cannot be deemed fully reliable. Given their potential weaknesses, it is crucial to identify more reproducible values, preferably ratios.

## 1. Introduction

Over recent decades, the number of patients undergoing Total Ankle Arthroplasty (TAA) has increased. The rising number of failed TAA has led to the development of revision implants, including modular ones, making these interventions feasible [1,2,3,4]. At the same time, other authors have explored reconstructions using customized tibial or talar components [5,6,7,8].

One of the primary goals of both primary and revision TAA is to restore the joint line (JL) to its original level, which is essential for optimal ankle function and to prevent complications such as joint instability, ankle pain, and reduced Range Of Motion (ROM) [2,9,10]. In primary TAA, achieving proper alignment of the prosthetic components, as well as good stability and a satisfactory ROM, is relatively straightforward since most bony and ligamentous landmarks are preserved [11,12]. However, in cases of trauma sequelae with severe Post-Traumatic Bone Loss (PTLB) or in revision surgery, some of these landmarks may no longer be identifiable, making the reconstruction of the JL to its original level more challenging [10].

A recent study proposed identifying the JL level using an adimensional ratio based on the talar dome and malleoli, but its application in complex TAA revisions is limited due to issues such as inadequate restoration of the fibula or medial malleolus length and significant coronal plane deformities [9]. Another method relies on absolute measurements to address coronal plane deformities, not considering factors like sex, BMI, or ankle size [13]. Consequently, the contralateral ankle is still the most widespread approach for planning complex TAA, as described in multiple studies [5,9,14]. However, planning this approach is complex as it involves obtaining a CT scan of the contralateral limb, which may not always be reproducible [5,6]. Moreover, it does not account for cases where the patient’s contralateral limb has also undergone anatomical changes due to trauma or previous surgical interventions such as TAA or arthrodesis.

The aim of this study was to evaluate the JL level in patients undergoing TAA due to severe PTBL or TAA revision using newly proposed reproducible anatomical landmarks and ratios described in the literature. The reproducibility and variability of the collected variables were assessed, and comparisons were made between the radiological parameters measured at the TAA and those at the contralateral ankle, while also exploring potential correlations.

## 2. Materials and Methods

A retrospective analysis was conducted on consecutive patients treated by two surgeons in our department from January 2018 to January 2024, following the principles outlined in the Declaration of Helsinki. Approval for this study was obtained from our University Institutional Review Board. Customized implants were designed and manufactured in collaboration with Adler Ortho (Adler Ortho SRL, Cormano, Milan, Italy).

The inclusion criteria involved patients who underwent customized TAA for severe PTBL or implant revision. Severe PTBL was defined as talar bone stock less than 50% or tibial bone stock loss precluding the use of standard components. Patients undergoing a two-stage revision for septic conditions were also included. At the same time, exclusion criteria included patients who underwent revision using standard primary TAA components.

Preoperative planning involved obtaining weight-bearing radiographs in two projections of both ankles, a full-length standing X-ray of the lower limbs, and an ankle CT scan according to manufacturer-established protocols to generate patient-specific 3D models.

CT scans covered the anterior tibial tuberosity through the mid-foot, with a spiral scan technique utilizing a 90° dorsiflexion angle (kV: 120, mA: 60) and a time rotation of 0.4–1 s. The field of view encompassed bone tissue, with a slice thickness of 0.6 mm and an overlapping slice interval.

Implant sizing was dictated by the anatomical considerations of the joint to be replaced. Custom-made implants were tailored to address both bone defects and provide adequate bone support. The design process employed GeoMagic Wrap 2017 (Artec 3D, Senningerberg, Luxemburg), a software tool designed to transform 3D scan data into usable models, integrating anatomical surfaces from affected and contralateral tibiotalar joints, as previously described [9].

Implant positioning aimed to restore the height of the talus, thus respecting the ligamentous isometry of both the calcaneofibular and the tibiocalcaneal ligament across the entire ROM. In cases of advanced talar osteonecrosis, a 9/10 talar component scale was available as an alternative if restoration of the talar space was not achievable during surgery, as suggested by Hussain et al. [15].

**Figure 1:**
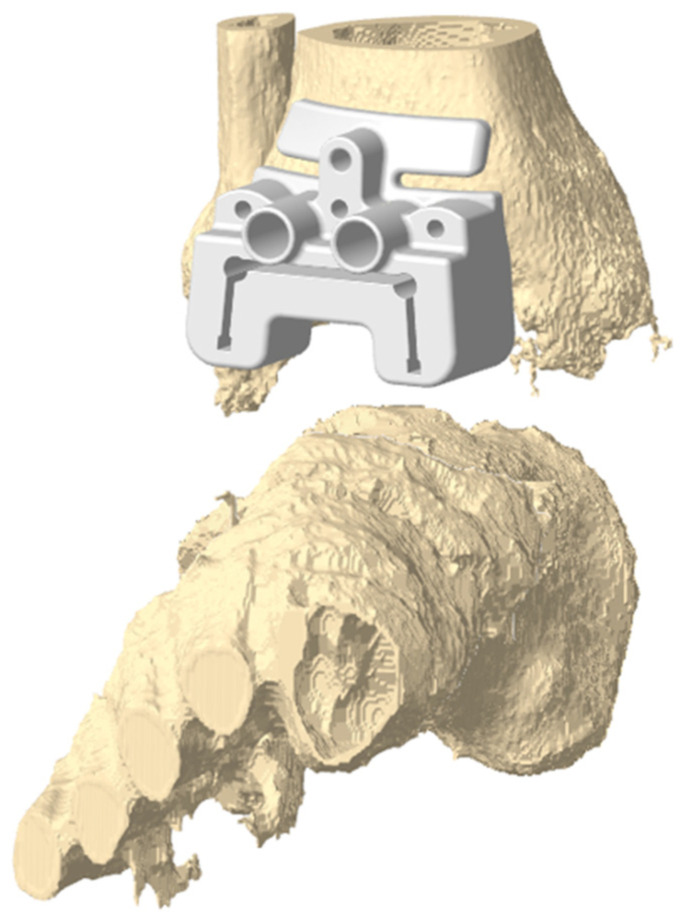
Example of PSI design for tibial cutting guides in patient number 7 using GeoMagic Control.

Once implant geometry and positioning were established, corresponding Patient-Specific Instruments (PSIs) were modeled on bone surfaces using GeoMagic Control 2017 (Figure 1).

For the radiological parameter evaluation, weight-bearing radiographs taken between 6 and 8 months postoperatively were employed, following the standard protocol at our department. The exam was conducted with the beam aligned directly perpendicular to the leg. All radiographs were checked to confirm they were taken using a standardized technique, focusing on accurate measurements along the true anteroposterior and latero-lateral lines of the ankle. A retrospective radiographic analysis was conducted to assess JL level in both anteroposterior and lateral views, with magnification errors corrected using implant stem or screw lengths for calibration. Three orthopedic surgeons collected radiological data independently, with a two-week interval between the first and second assessments, to assess the reproducibility of the data.

**Figure 2:**
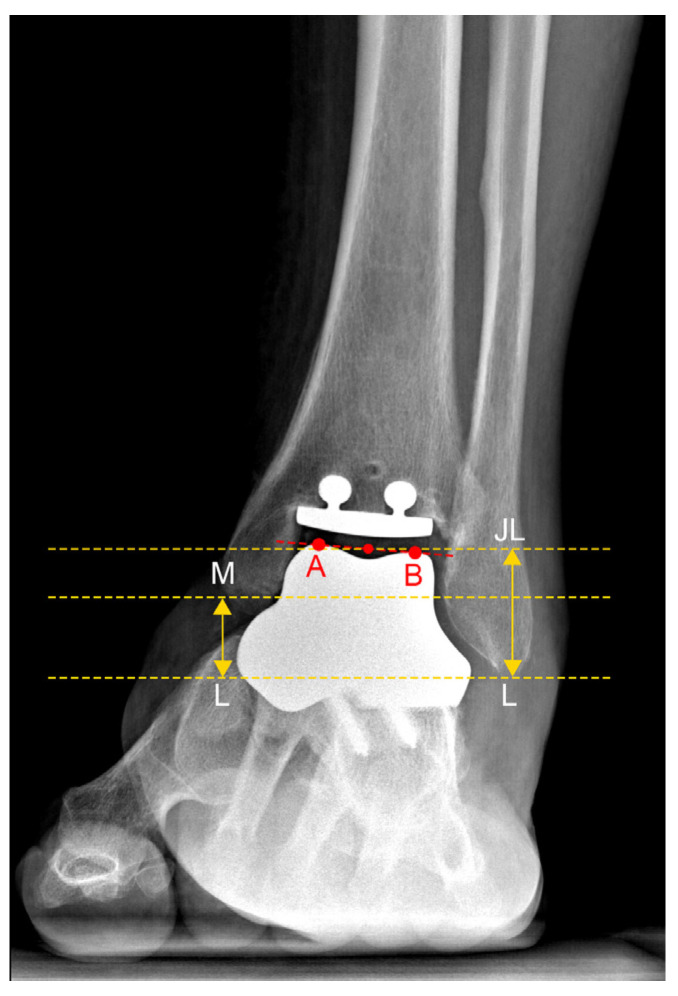
Joint Line Height Ratio (JLHR) in patient n. 4, who underwent custom-made TAA for talar osteonecrosis: The lateral malleolus tip (L) and posterior colliculus of the medial malleolus (M) were labeled as points. Two parallel lines were drawn passing from points L and M, and the distance between these lines was measured as the vertical intermalleolar distance (M-L). The most medial (A) and lateral (B) points of the talar dome were also identified. The line AB connecting these points was measured for distance. The midpoint of line AB (JL) represented the center of the ankle joint. A third line, parallel to the ground and passing through the JL point, was drawn. The distance between the line passing through point JL and point L (JL-L) was measured as the vertical joint line distance. The ratio of vertical joint line distance (JL-L) to vertical intermalleolar distance (M-L) was calculated as the JLHR.

**Figure 3:**
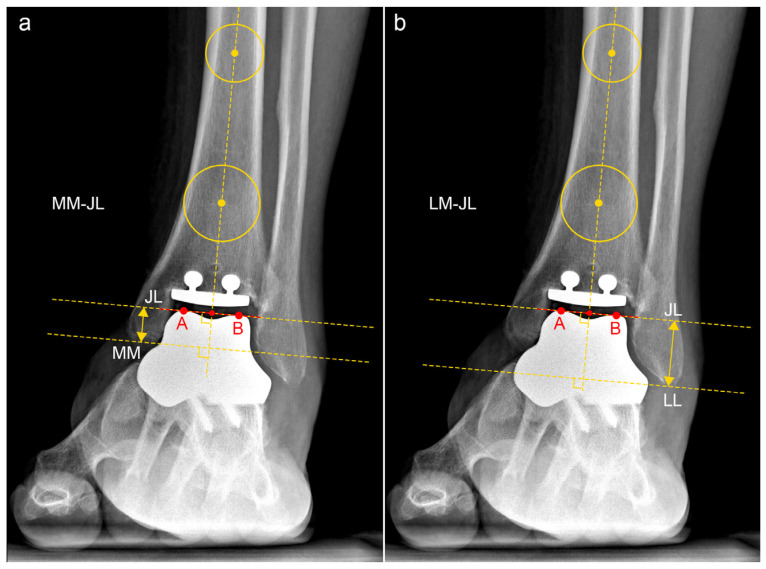
Medial Malleolus–Joint Line (MM-JL) and Lateral Malleolus–Joint Line (LM-JL) distances in patient n. 4, who underwent custom-made TAA for talar osteonecrosis: (**a**) The tibial anatomical axis was determined by drawing two circles 5 and 10 cm proximally from the joint line. The tibial anatomical axis passes through these two points. The posterior colliculus of the medial malleolus was identified as point MM. The most medial and lateral points of the talar dome were labeled as points A and B, respectively. The distance between points A and B (line AB) was measured. The midpoint of line AB (JL) represented the center of the ankle joint. The distance between MM and JL was calculated as the distance between two parallel lines perpendicular to the anatomical tibial axis passing through these two specified points. (**b**) The tibial anatomical axis was determined by drawing two circles 5 and 10 cm proximally from the joint line. The tibial anatomical axis passes through these two points. The tip of the lateral malleolus was identified as point LM. The most medial and lateral points of the talar dome were labeled as points A and B, respectively. The distance between points A and B (line AB) was measured. The midpoint of line AB (JL) represented the center of the ankle joint. The distance between LM and JL was calculated as the distance between two parallel lines perpendicular to the anatomical tibial axis passing through these two specified points.

In the anteroposterior view, the JL was defined as the midpoint of the talar dome, with distances calculated perpendicular to the ground line. The parameters calculated included the following:

- JL Height Ratio (JLHR) post TAA as described by Harnroongroj et al. [9] (Figure 2).
- The distance between the tip of the posterior colliculus of the medial malleolus and the JL (MM-JL) (Figure 3a).
- The distance between the apex of the peroneal malleolus and the JL (LM-JL) (Figure 3b).

**Figure 4:**
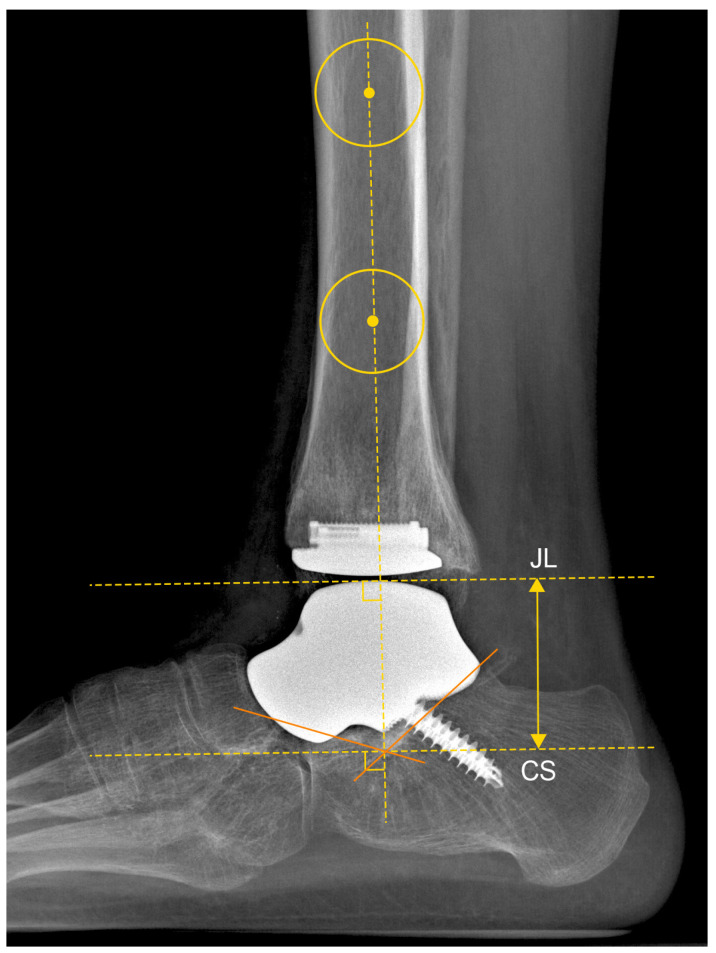
Calcaneus Sulcus–Joint Line (CS-JL) distance in patient n. 4, who underwent custom-made TAA for talar osteonecrosis: The tibial anatomical axis was determined by drawing two circles 5 and 10 cm proximally from the joint line. The tibial anatomical axis passes through these two points. The Calcaneus Sulcus (CS) was identified as the intersection of a line drawn along the superior surfaces of the anterior process and the posterior facet of the calcaneus. The Joint Line (JL) is identified as the apex of the talar dome with the tibiotalar angle set at 90°. The distance between CS and JL was calculated as the distance between two parallel lines perpendicular to the anatomical tibial axis passing through these two specified points.

In the latero-lateral view, the parameter calculated was the Calcaneus Sulcus–JL distance (CS-JL) (Figure 4).

**Figure 5:**
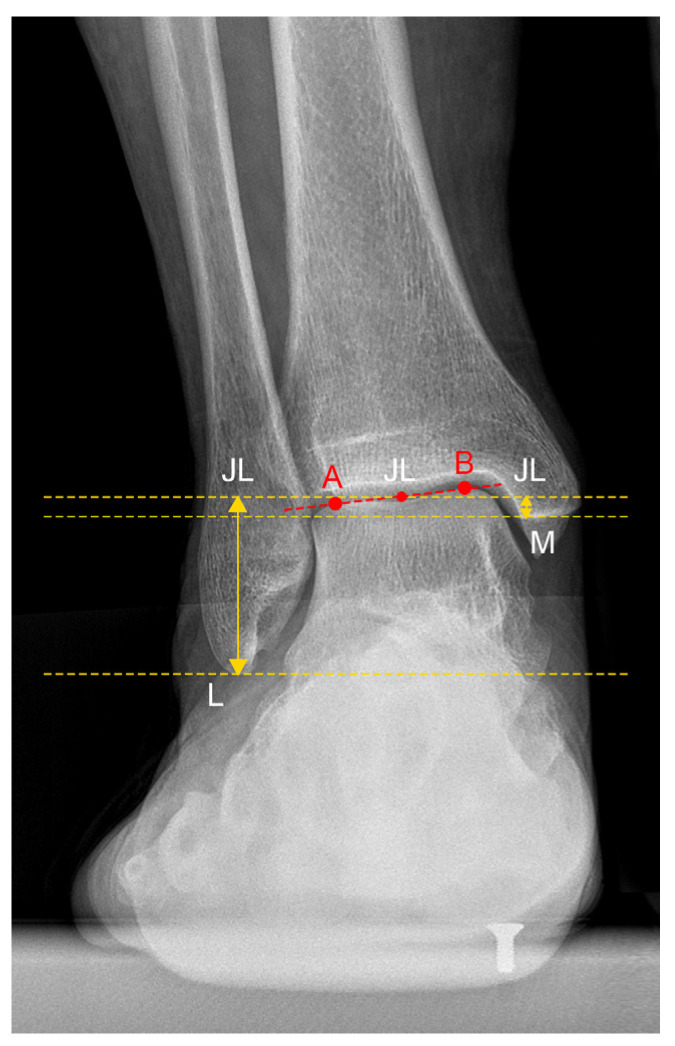
Joint Line Height Ratio (JLHR) on the contralateral healthy side of patient n. 6, as previously described in Figure 1. The most medial and lateral points of the talar dome were labeled as points A and B, respectively. The midpoint of line AB (JL) represented the center of the ankle joint. The intermalleolar distance was obtained as the difference between the Joint Line–Lateral Malleolus distance (JL-L) and the Joint Line–Medial Malleolus distance (JL-M).

**Figure 6:**
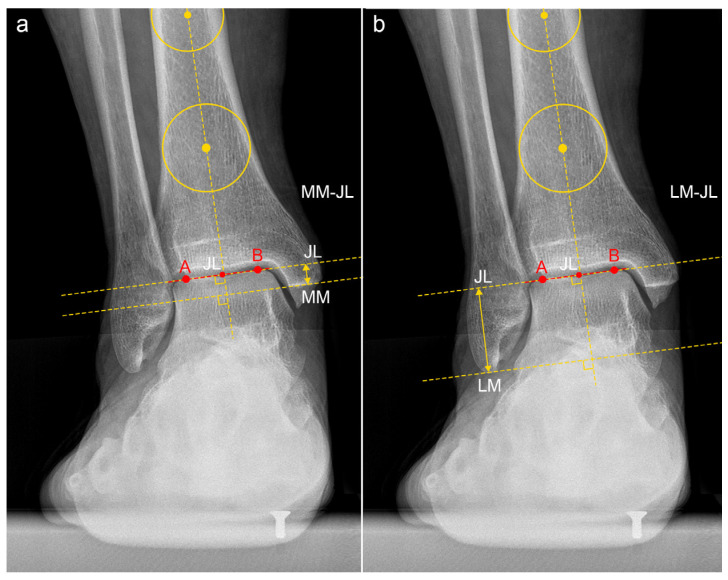
(**a**) Medial Malleolus–Joint Line (MM-JL) and (**b**) Lateral Malleolus–Joint Line (LM-JL) distances on the contralateral healthy side of patient n. 6, as previously described in Figure 2. The most medial and lateral points of the talar dome were labeled as points A and B, respectively. The midpoint of line AB (JL) represented the center of the ankle joint.

**Figure 7:**
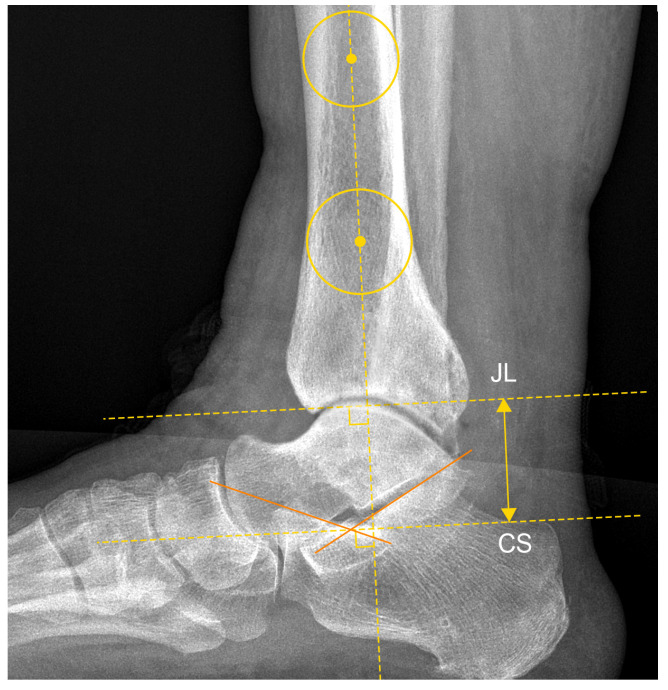
Calcaneus Sulcus–Joint Line (CS-JL) distance on the contralateral healthy side of patient n. 6, as previously described in Figure 3.

A comparison of JL level after TAA was performed in relation to the preoperative X-ray of the contralateral side, from which the same parameters were collected (Figure 5, Figure 6 and Figure 7).

### Statistical Analysis

Intraobserver reliability was assessed using intraclass correlation coefficients (ICCs), with a Cronbach’s alpha exceeding 0.80 signifying perfect reliability, while interobserver reliability was investigated using Cohen’s kappa coefficient. Radiological parameter variables were calculated by averaging the values collected by the three orthopedic surgeons independently, and they were reported as mean values, standard deviations (SD), and ranges. The Coefficient of Variation (CV = SD/mean*100), as a percentage, was calculated to verify the lower variability among the collected variables. The normality of the continuous variables’ data distribution was evaluated using the Shapiro–Wilk test. Paired T-tests were conducted for normally distributed data to compare customized TAA and contralateral ankle parameters. Possible correlations between the analyzed parameters were investigated using Pearson’s test. Statistical significance was defined as *p* < 0.05. Statistical analyses were performed using Jamovi software (The Jamovi project—jamovi Version 1.6, 2021).

## 3. Results

### 3.1. Patient Demographics

Thirteen patients underwent customized TAA for severe PTBL or implant revision between January 2018 and January 2024. Among them, nine were male, with an average age of 52.4 ± 9.88 years at the time of surgery.

Nine patients had post-traumatic etiology with severe bone loss, while four underwent prosthetic revision. One of the patients undergoing revision had aggressive juvenile rheumatic pathology, for which bilateral knee prosthetic replacement and a contralateral TAA had also been performed. The other three patients, diagnosed with septic loosening, required a multistep therapy that included employing a cemented spacer and targeted antibiotic treatment until imaging and laboratory parameters normalized. Demographic information is summarized in Table 1.

### 3.2. Complications

One patient (Patient No. 6) experienced an intraoperative complication, an incomplete iatrogenic lateral malleolar fracture, which was managed with the insertion of 2 K-wires. The nondisplaced nature of the fracture and its stability during joint mobility tests, allowed for this type of treatment.

### 3.3. Reliability and Coefficient of Variation Assessment

After collecting the radiological parameters, intraobserver reliability demonstrated ICC values of 0.92, 0.88, and 0.93 for the three orthopedic surgeons, respectively, while interobserver reliability showed a kappa coefficient of 0.87. The Shapiro–Wilk test revealed no significant deviation from a normal distribution. The lowest Coefficient of Variation values were observed for the CS-JL distance, LM-JL distance, and CS-JL distance of the contralateral limb. The radiological parameters are summarized in Table 2.

### 3.4. Comparison between Customized TAA and Contralateral Ankle

**Figure 8:**
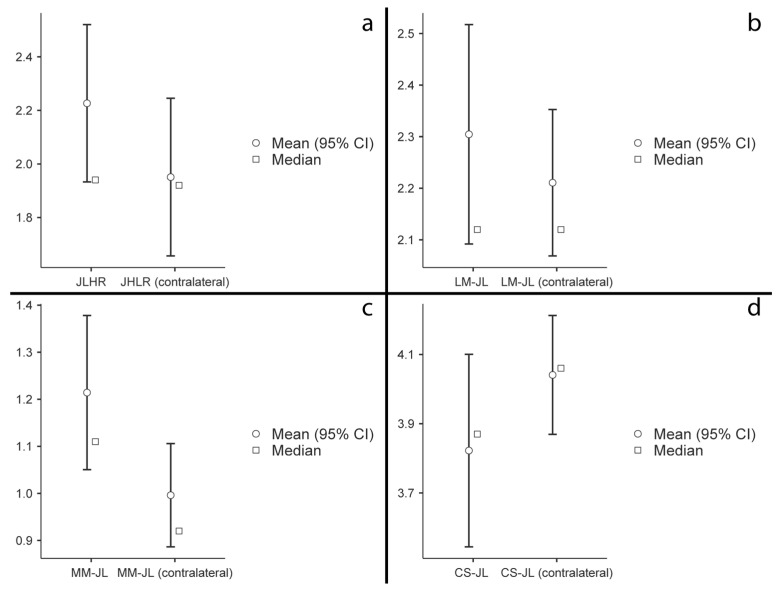
Paired Student’s *t*-test comparing the postoperative radiological parameters with those of the contralateral limb: (**a**) JLHR = Joint Line Height Ratio; (**b**) LM-JL = Lateral Malleolus–Joint Line distance; (**c**) MM-JL = Medial Malleolus–Joint Line distance; (**d**) CS-JL = Calcaneus Sulcus–Joint Line distance.

The paired Student’s *t*-test conducted to compare the JL values obtained postoperatively with those of the contralateral limb did not show a significant difference for any of the analyzed variables (Figure 8).

### 3.5. Correlation Analysis

**Figure 9:**
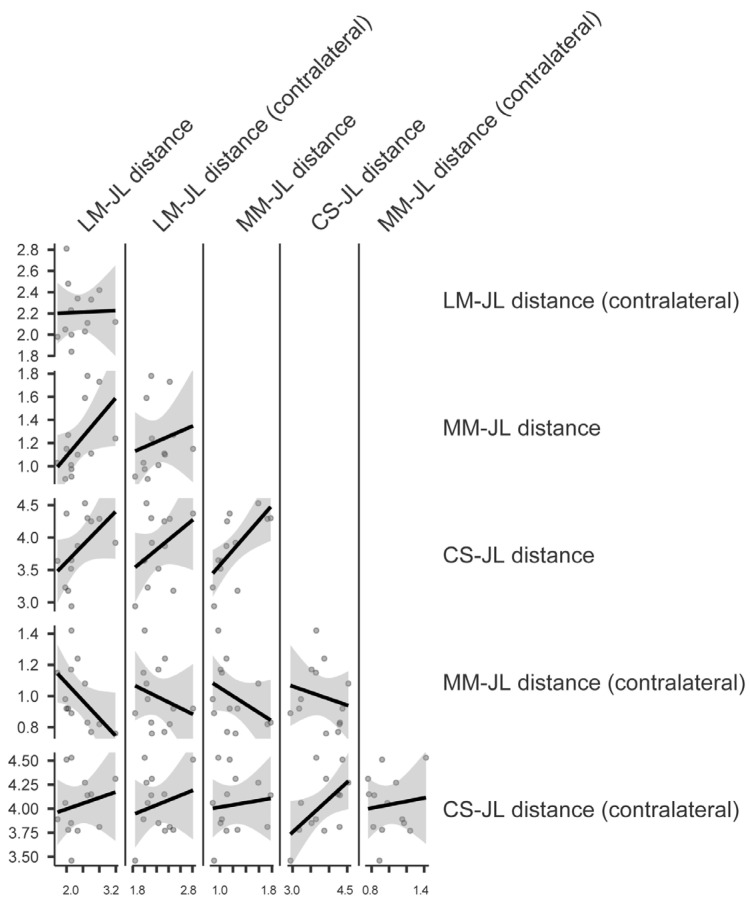
Pearson linear correlation plot: LM-JL = Lateral Malleolus–Joint Line distance; MM-JL = Medial Malleolus–Joint Line distance; CS-JL = Calcaneus Sulcus–Joint Line distance.

A statistically significant correlation was observed between the CS-JL and MM-JL distances of the limb undergoing TAA, as well as between the CS-JL of the operated limb and that of the contralateral ankle (Table 3) (Figure 9).

## 4. Discussion

In cases of TAA for severe PTBL or TAA revision, the primary surgical objectives are to restore the original anatomy, regain function, and provide a stable joint [16]. However, achieving these goals can be challenging, particularly in patients with significant anatomical variations. As a result, determining the correct positioning of the JL level is a significant challenge for orthopedic surgeons, as there are currently no standardized values to guide this decision [17].

Given the complexity of the topic and the limited studies available in the literature [9,13,18], leveraging experiences and analogous concepts from similar areas has provided valuable insights. Restoration of the JL has been advocated as a crucial principle in Total Knee Arthroplasty (TKA), as it has been associated with improved clinical outcomes and enhanced ROM [13,19,20]. Conversely, an elevated JL in TKA can lead to restricted ROM, increased patellofemoral issues, and overall poorer clinical results [13,21,22]. Similarly, restoring the native JL of the ankle joint may also contribute to improved ROM and clinical outcomes in patients undergoing TAA, as it not only impacts the tibiotalar ligaments but also affects the leverage and excursion lengths of the flexor and extensor tendons of the foot [13].

A recent study proposed identifying the JL level using an adimensional ratio based on the talar dome and malleoli, but this method has limitations in complex cases, such as post-traumatic patients with inadequate restoration of fibula or medial malleolus length and significant coronal plane deformities [9]. Furthermore, while easily obtainable with an anteroposterior radiograph, it is not calculable intraoperatively. Another proposed method employs four absolute measurements to address coronal plane deformities. Nonetheless, some limitations arise, as it does not account for demographic factors like sex, BMI, and ankle size, nor does it include standardized radiographic markers, complicating reliance on the data [13].

Thus, in most cases, the contralateral ankle is still preferred as a reference for establishing the correct JL level [5,9,14]. However, this method also has limitations due to the need for additional radiological examinations and the unavailability of the contralateral limb as a model in certain cases, such as when the patient has previously undergone TAA or ankle arthrodesis on the contralateral ankle.

In other joint prosthetic revisions, attempts have been made to identify anatomical landmarks or ratios at the prosthesis level to restore JL level without relying on the contralateral limb. For example, in TKA, various bony marks have been utilized [19,23,24]. However, to date, the most reliable parameters internationally recognized are ratios [23,25,26,27]. Notably, absolute measurements of anatomical landmarks, such as the distance from the epicondyles to the knee JL, have shown significant variability between individuals and genders. In contrast, ratios, such as the epicondylar ratio (distance from the lateral epicondyle to the knee JL relative to femoral width), exhibit no significant gender difference [23]. In this study, we aimed to evaluate the JL level in patients undergoing TAA due to severe PTBL or TAA revision using newly proposed reproducible anatomical landmarks and ratios described in the literature [9].

The newly proposed radiological parameters are based on absolute distances from commonly used anatomical landmarks in preoperative evaluations. The target was to provide surgeons with guidelines that are easy to apply in clinical practice and can also be estimated intraoperatively. Although the results did not allow for definitive conclusions regarding the proposed landmarks, or the previously described ratio [9], some positive aspects can still be highlighted.

The intraobserver and interobserver reliability observed in our study was excellent, consistent with previous findings for both ratio measurements, such as JLHR, and absolute values, such as the method proposed by Fletcher [9,13]. However, these measurements require experience, especially in identifying anatomical landmarks surrounded by osteophytes in post-traumatic patients.

While our study did not allow for definitive conclusions, trends were observed, including an increase in MM-JL, indicating JL proximalization compared with the contralateral ankle. However, this elevation of the JL did not correlate with a talus height increase; in fact, CS-JL values were lower than those of the contralateral ankle. This corroborates previous observations by other authors, indicating that ankle osteoarthritis is associated with JL elevation [9,28,29,30]. It is also interesting to note that this JL proximalization, not being compensated by a higher talar component compared with the contralateral side, leads to an absolute lower limb shortening.

Further analysis revealed an interesting correlation between MM-JL and CS-JL: an increase in one correlated with an increase in the other. This could be interpreted as a proportionality between talus height and medial malleolus dimensions. However, we believe this finding could also suggest that attempting to restore talus height may not be the correct strategy for JL reconstruction, as a higher talus often correlates with a greater JL distance from the medial malleolus, causing further proximalization of the JL.

Among the parameters analyzed in this study, none can currently be considered reliable for JL positioning in cases of trauma sequelae with severe bone loss or in revision surgery. While JLHR appeared theoretically reliable, its high coefficient of variation and wide range in our case series suggest its limitations. Regarding absolute values, a potentially useful parameter could be the average 1 cm between JL and the posterior colliculus of the medial malleolus, referred to as MM-JL, found in the healthy contralateral ankles. However, this has limitations in cases requiring malleolar osteotomies, in patients with severe post-traumatic deformities, and for the variability related to different sex, ankle size, etc.

In TKA, parameters based on JL distances from anatomical structures have gradually been replaced by various ratios [23,26,27,28]. This approach accounts for variability associated with sex, ankle size, and BMI, suggesting its potential suitability for complex TAA cases as well.

Despite the evident challenges, restoring the correct JL level may play a crucial role in the future of TAA. When particular attention is given to restoring the JL level, postoperative results show an increase in ROM, especially dorsiflexion, by up to 6.3 degrees [13], and this improvement was also associated with better patient-reported outcomes [13,18].

This study presents some limitations. The small number of patients does not allow for exhaustive conclusions; however, the case series of custom-made TAA is one of the largest ever reported [5,6,8]. Radiological parameters of the JL were not correlated with any clinical outcome; thus, their actual influence cannot be determined. However, it should be noted that to date, the only two studies utilizing parameters for JL measurement have not measured the difference in ROM between pre- and postoperative stages in individual patients; therefore, the observed changes could be due to a heterogeneous population already at the preoperative stage. Finally, while JL is a particularly important parameter for the biomechanics of the tibiotalar joint, other parameters, such as its anteroposterior positioning on the coronal plane or its version on the axial plane, have not been taken into account in this or other studies currently available on ankle prosthetics. Only a careful understanding of the kinetic demands of a post-traumatic ankle can help us improve the design and positioning of TAA in the future.

## 5. Conclusions

This study demonstrated good reproducibility of the parameters used to evaluate the JL in TAA for patients with severe PTBL or undergoing revision surgery. Although no statistically significant differences were found compared with the contralateral ankle, a trend toward JL proximalization was noted. A significant correlation between CS-JL and MM-JL distances in the operated limb was also observed, warranting further investigation. The importance of restoring the JL level could have significant clinical implications, both for improving postoperative ROM and enhancing PROMs. Given these results, the parameters analyzed cannot be considered fully reliable for determining the JL in cases of severe PTBL or revision surgery. However, this study provides valuable insights by highlighting potential weaknesses in both previously described ratios and the newly proposed parameters based on absolute radiological distances. Identifying more reproducible values, preferably ratios, could better accommodate variability related to factors like sex, ankle size, or BMI, potentially improving outcomes in complex TAA cases. Despite some limitations, these findings offer promising directions for future research and clinical practice enhancements.

## Figures and Tables

**Table 1:** Patient demographics.

Patients	Age	Sex	Surgical indication
1	56	M	Post-traumatic
2	57	F	Implant revision (septic loosening)
3	50	M	Post-traumatic
4	45	M	Post-traumatic
5	30	M	Post-traumatic
6	58	M	Implant revision (septic loosening)
7	42	M	Post-traumatic
8	49	F	Post-traumatic
9	69	F	Implant revision (aseptic loosening)
10	49	M	Post-traumatic
11	58	M	Implant revision (septic loosening)
12	62	F	Post-traumatic
13	56	M	Post-traumatic

**Table 2:** Radiological variables with the respective coefficient of variation.

Radiological Parameter	Mean ± Standard Deviation	Median	Range	Coefficient of Variation (%)
JLHR ^1^	2.23 ± 0.54	1.94	1.64–3.44	24.3%
LM-JL ^2^ distance	2.30 ± 0.39 cm	2.12 cm	1.78–3.19 cm	17.0%
MM-JL ^3^ distance	1.21 ± 0.30 cm	1.11 cm	0.89–1.78 cm	24.9%
CS-JL ^4^ distance	3.82 ± 0.51 cm	3.87 cm	2.94–4.53 cm	13.4%
JLHR ^1^ (contralateral)	1.95 ± 0.54	1.92	1.49–3.54	27.7%
LM-JL ^2^ distance (contralateral)	2.21 ± 0.26 cm	2.12 cm	1.84–2.81 cm	11.8%
MM-JL ^3^ distance (contralateral)	1.00 ± 0.20 cm	0.92 cm	0.76–1.42 cm	20.3%
CS-JL ^4^ distance (contralateral)	4.04 ± 0.32 cm	4.06 cm	3.46–4.53 cm	7.8%

^1^ JLHR = Joint Line Height Ratio; ^2^ LM-JL = Lateral Malleolus–Joint Line; ^3^ MM-JL = Medial Malleolus–Joint Line; ^4^ CS-JL Calcaneus Sulcus–Joint Line.

**Table 3:** Pearson linear correlation matrix.

		LM-JL ^1^	MM-JL ^2^	CS-JL ^3^	LM-JL ^1^ (Contralateral)	MM-JL ^2^ (Contralateral)
MM-JL ^2^	Pearson R	0.546	—		0.195	
	*p*-value	0.054	—		0.524	
CS-JL ^3^	Pearson R	0.497	0.679	—	0.387	
	*p*-value	0.084	0.011 *	—	0.191	
LM-JL ^1^ (contralateral)	Pearson R	0.026	0.195	0.387	—	
	*p*-value	0.933	0.524	0.191	—	
MM-JL ^2^ (contralateral)	Pearson R	−0.547	−0.398	−0.205	−0.245	—
	*p*-value	0.053	0.177	0.502	0.420	—
CS-JL ^3^ (contralateral)	Pearson R	0.183	0.110	0.561	0.207	0.112
	*p*-value	0.549	0.722	0.046 *	0.497	0.716

^1^ LM-JL = Lateral Malleolus–Joint Line distance; ^2^ MM-JL = Medial Malleolus–Joint Line distance; ^3^ CS-JL = Calcaneus Sulcus–Joint Line distance; * = statistically significative value (*p* < 0.05).

## Data Availability

The clinical data from this study are available upon request to the corresponding author.
